# Supplementary material for: Role and mechanism of NCAPD3 in promoting malignant behaviors in gastric cancer
Source: Front Pharmacol. 2024 Apr 22;15:1341039. doi: 10.3389/fphar.2024.1341039 (PMC11070777; doi:10.3389/fphar.2024.1341039)
Supplement: Supplementary file 11 [file DataSheet2.ZIP › GSEA/GO_CC/my_analysis.Gsea.1599461408869/GO_CONTRACTILE_FIBER.html]

Details for gene set GO\_CONTRACTILE\_FIBER[GSEA]

|  || Dataset | filtered\_dataset.sample\_info.cls#WT\_versus\_NCAPD3\_MUT |
| Phenotype | sample\_info.cls#WT\_versus\_NCAPD3\_MUT |
| Upregulated in class | NCAPD3\_MUT |
| GeneSet | GO\_CONTRACTILE\_FIBER |
| Enrichment Score (ES) | -0.48841575 |
| Normalized Enrichment Score (NES) | -2.2468264 |
| Nominal p-value | 0.0025839794 |
| FDR q-value | 0.015152376 |
| FWER p-Value | 0.023 |
Table: GSEA Results Summary

  

Fig 1: Enrichment plot: GO\_CONTRACTILE\_FIBER      
 Profile of the Running ES Score & Positions of GeneSet Members on the Rank Ordered List

  

| SYMBOL | TITLE | RANK IN GENE LIST | RANK METRIC SCORE | RUNNING ES | CORE ENRICHMENT || 1 | 7170 | TPM3 | 24 | 1.018 | 0.0588 | No |
| 2 | 9759 | HDAC4 | 46 | 0.925 | 0.1129 | No |
| 3 | 9456 | HOMER1 | 198 | 0.680 | 0.0552 | No |
| 4 | 805 | CALM2 | 423 | 0.518 | -0.0672 | No |
| 5 | 801 | CALM1 | 424 | 0.518 | -0.0285 | No |
| 6 | 1605 | DAG1 | 823 | 0.304 | -0.2919 | No |
| 7 | 6385 | SDC4 | 871 | -0.282 | -0.3046 | No |
| 8 | 667 | DST | 886 | -0.303 | -0.2920 | No |
| 9 | 2317 | FLNB | 936 | -0.360 | -0.3003 | No |
| 10 | 7168 | TPM1 | 1086 | -0.465 | -0.3727 | No |
| 11 | 114907 | FBXO32 | 1248 | -0.625 | -0.4417 | Yes |
| 12 | 286 | ANK1 | 1284 | -0.672 | -0.4167 | Yes |
| 13 | 100820829 | MYZAP | 1291 | -0.682 | -0.3700 | Yes |
| 14 | 7414 | VCL | 1308 | -0.712 | -0.3283 | Yes |
| 15 | 6262 | RYR2 | 1323 | -0.741 | -0.2830 | Yes |
| 16 | 10611 | PDLIM5 | 1335 | -0.758 | -0.2343 | Yes |
| 17 | 4622 | MYH4 | 1355 | -0.811 | -0.1874 | Yes |
| 18 | 6840 | SVIL | 1377 | -0.916 | -0.1340 | Yes |
| 19 | 6711 | SPTBN1 | 1381 | -0.925 | -0.0670 | Yes |
| 20 | 493 | ATP2B4 | 1401 | -1.166 | 0.0065 | Yes |
Table: GSEA details [plain text format]

  

Fig 2: GO\_CONTRACTILE\_FIBER      
 Blue-Pink O' Gram in the Space of the Analyzed GeneSet

  

Fig 3: GO\_CONTRACTILE\_FIBER: Random ES distribution      
 Gene set null distribution of ES for **GO\_CONTRACTILE\_FIBER**
